# Supplementary figures and images for: SigmoID: a user-friendly tool for improving bacterial genome annotation through analysis of transcription control signals
Source: PeerJ. 2016 May 24;4:e2056. doi: 10.7717/peerj.2056 (PMC4888284; doi:10.7717/peerj.2056)

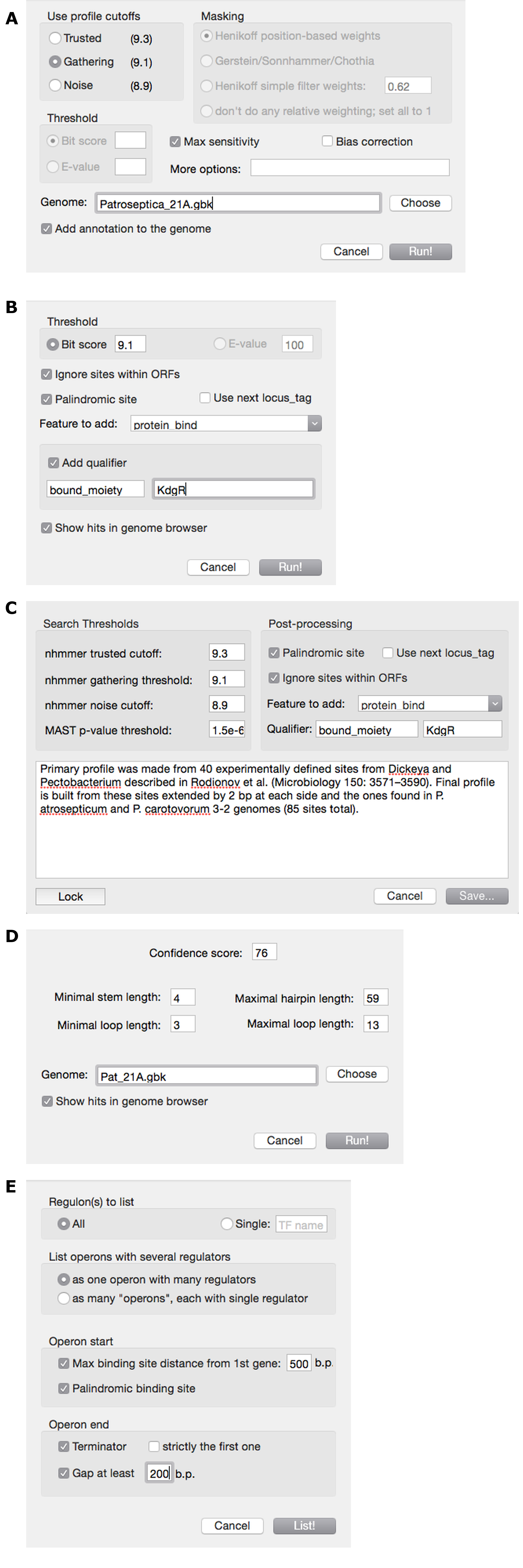

Supplement: Figure S1 — (A) nhmmer configuration window; (B) nhmmer post-processing script configuration window; (C) Profile Wizard window; (D) TransTerm HP configuration window; (E) “List regulons...” function settings window. [file peerj-04-2056-s005.png]
