# Supplementary material for: SigmoID: a user-friendly tool for improving bacterial genome annotation through analysis of transcription control signals
Source: PeerJ. 2016 May 24;4:e2056. doi: 10.7717/peerj.2056 (PMC4888284; doi:10.7717/peerj.2056)

1. ArcA

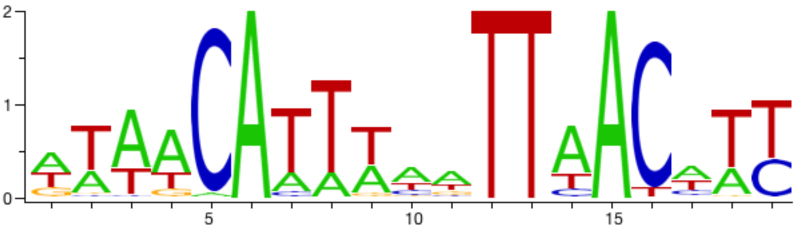

2. ArgR

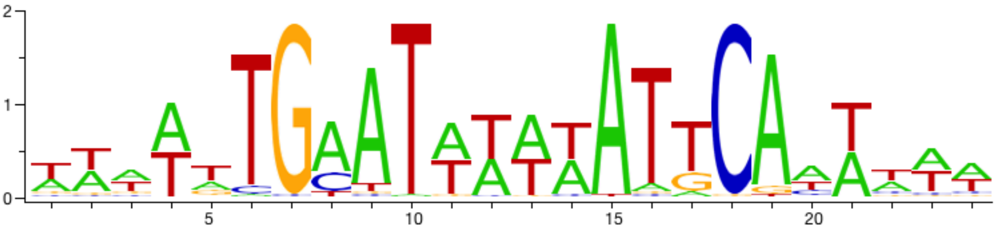

3. AscG

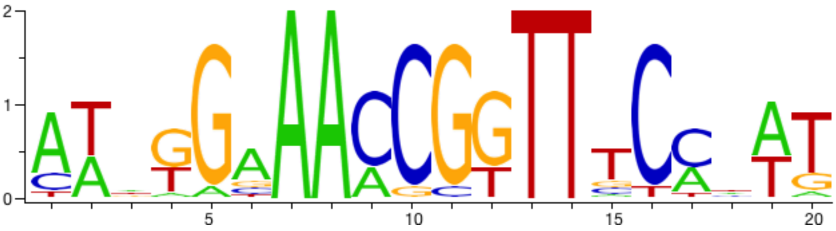

4. BaeR

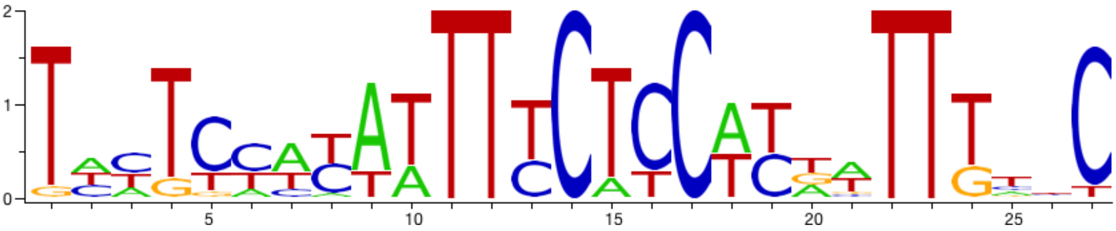

5. CpxR

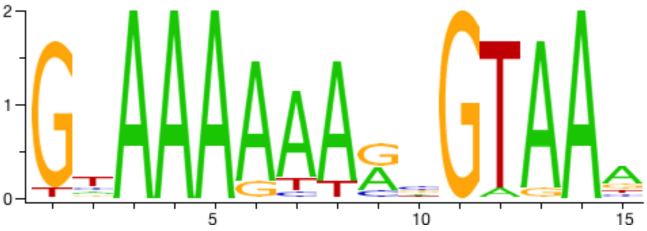

6. CRP

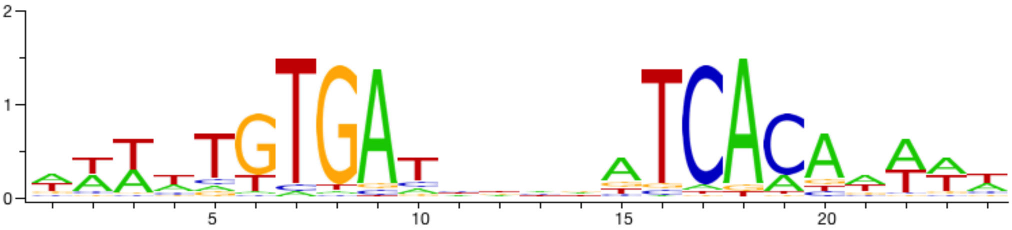

7. FliA

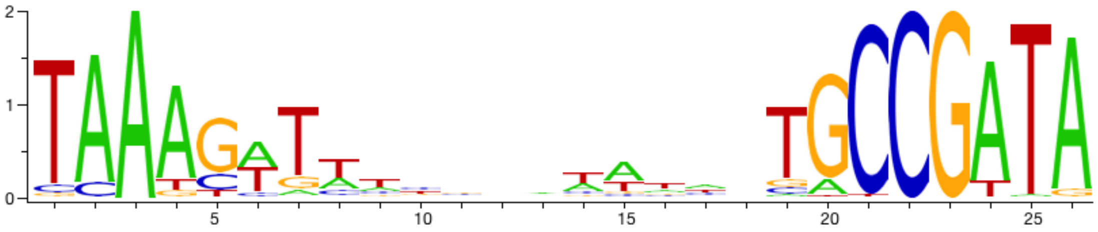

8. FNR

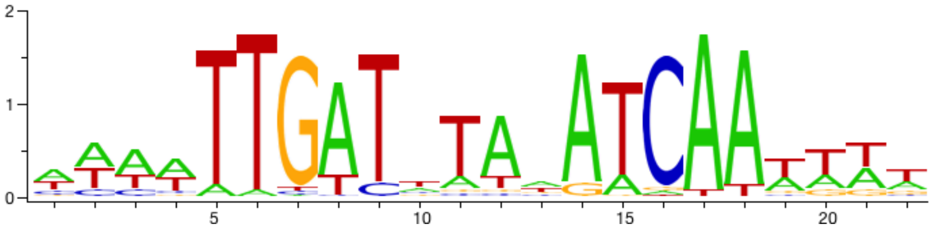

9. FruR

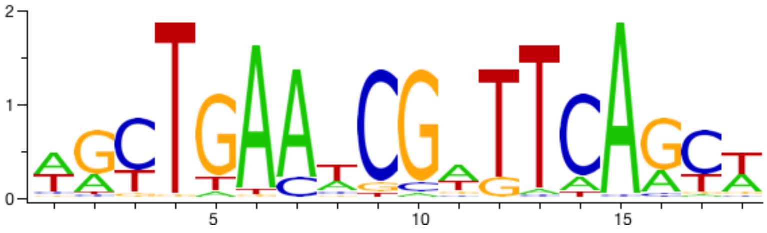

10. GalR

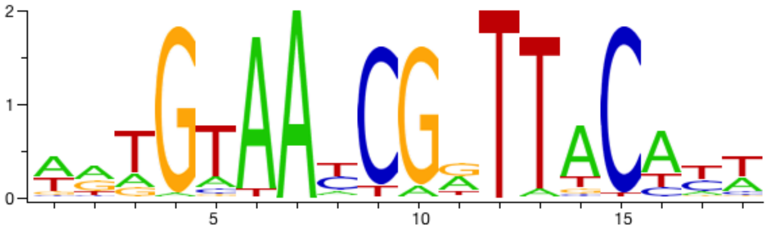

11. GlrR

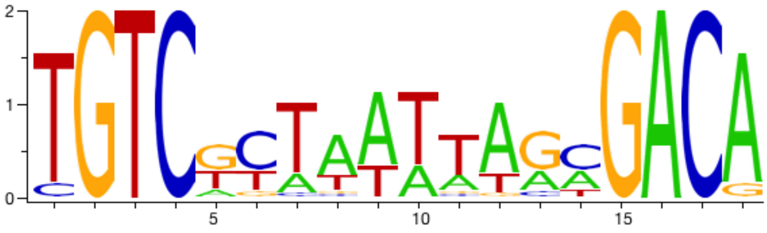

12. HrpL

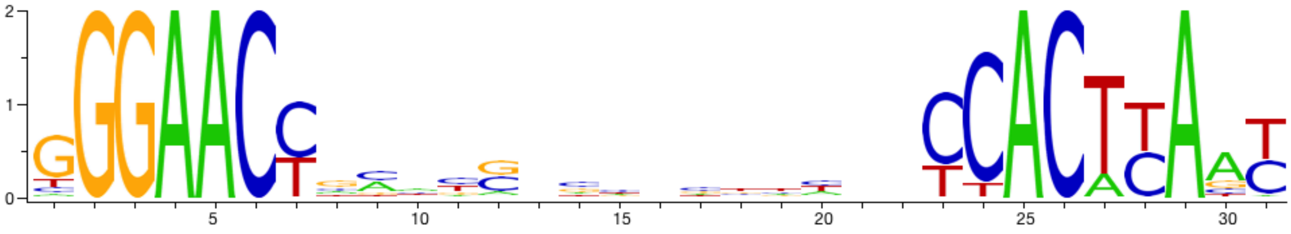

13. HrpS

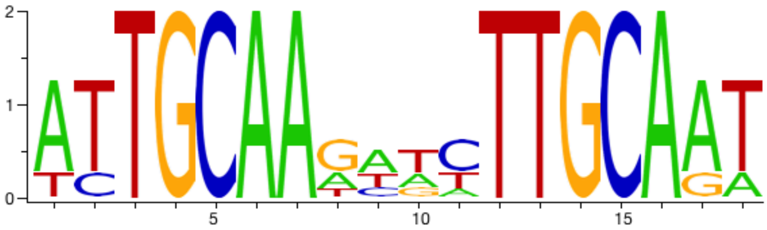

14. HyfR

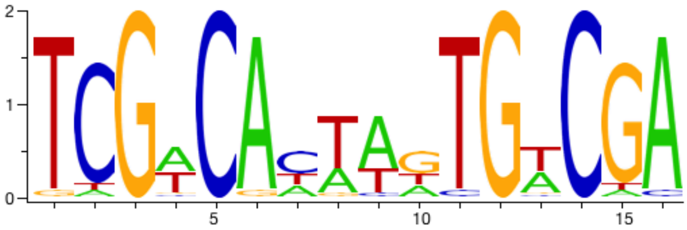

15. KdgR

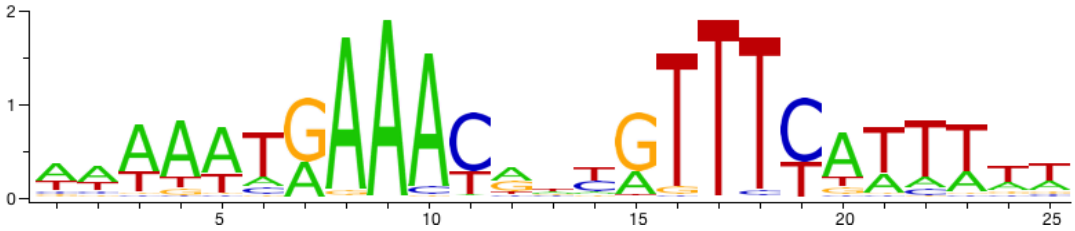

16. LexA

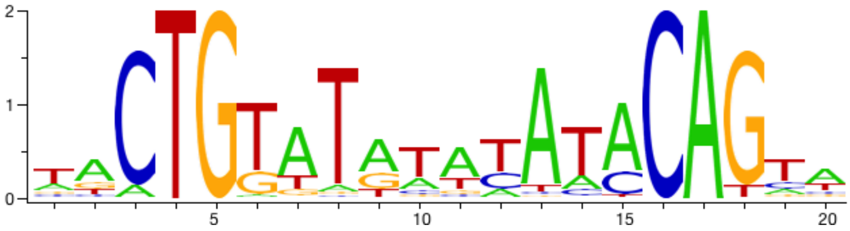

17. MetJ

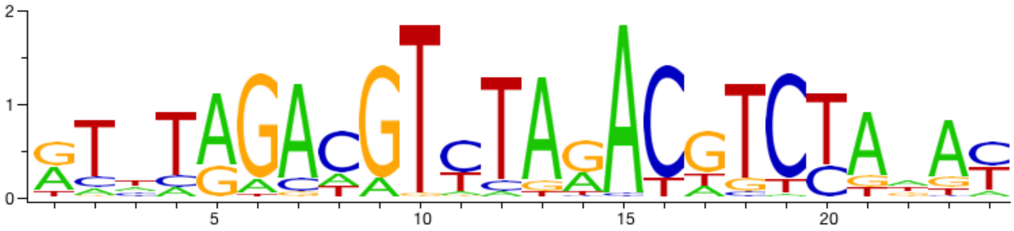

18. MetR

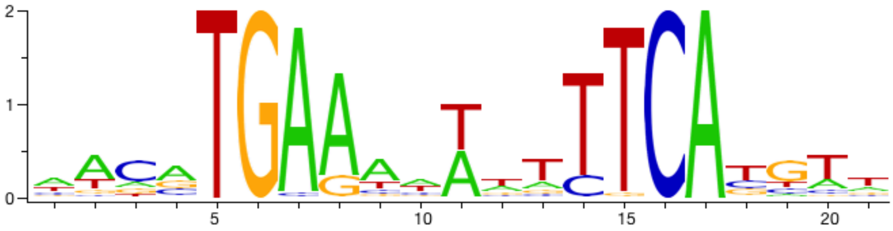

19. NarP

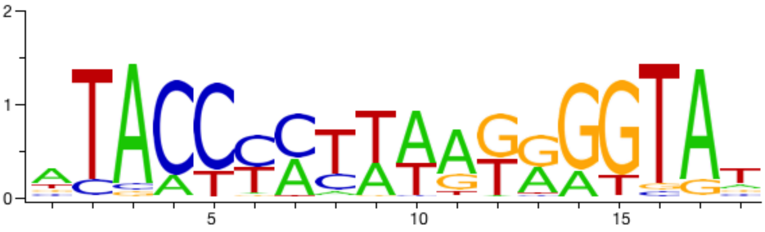

20. NifA

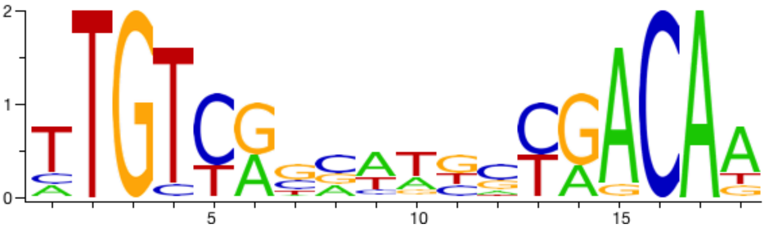

21. NorP

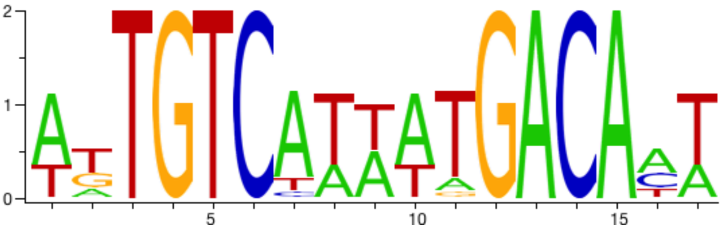

22. NsrR

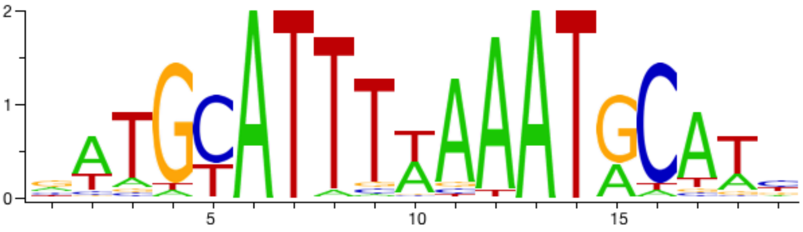

23. NtrC

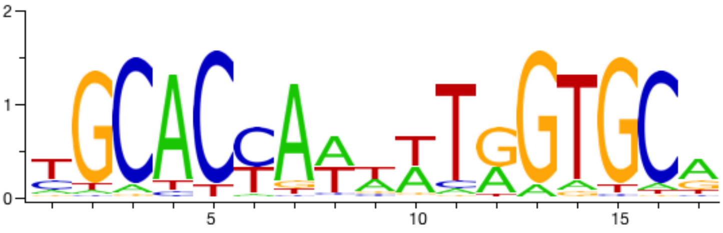

24. OxyR

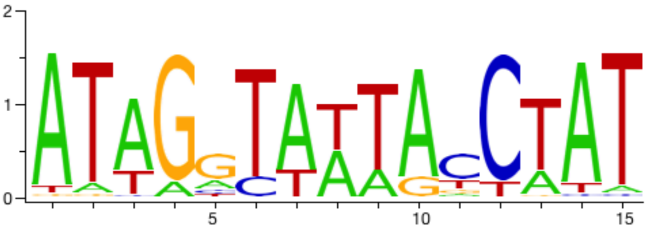

25. PgrR

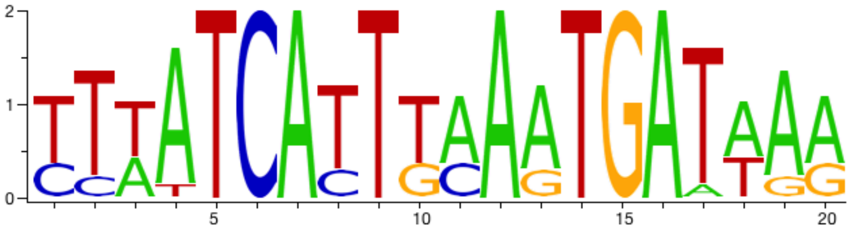

26. PhoB

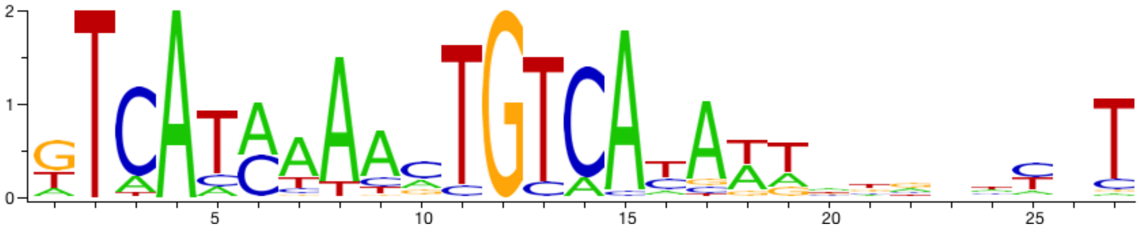

27. PhoP1

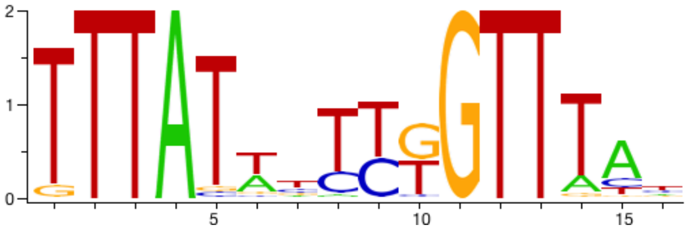

28. PhoP2

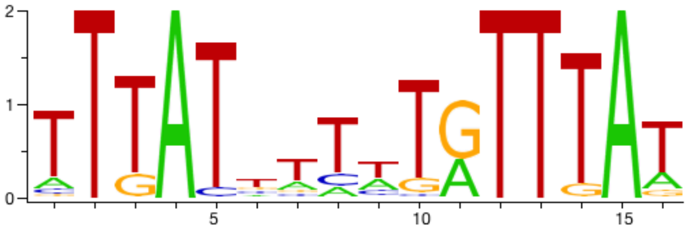

29. PmrA

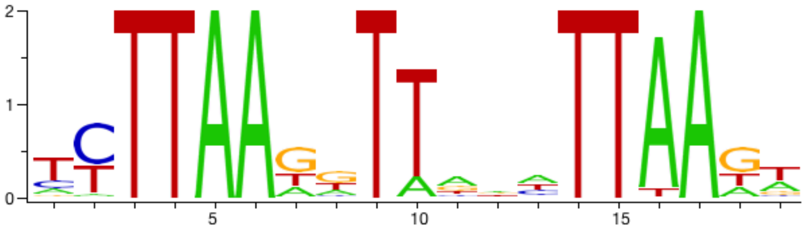

30. PspF

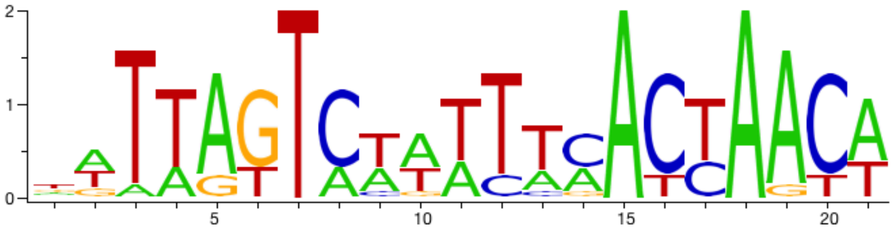

31. PurP

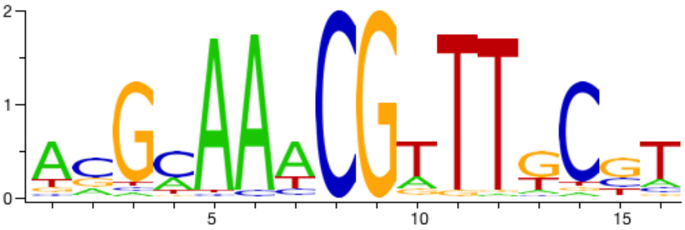

32. RcsB

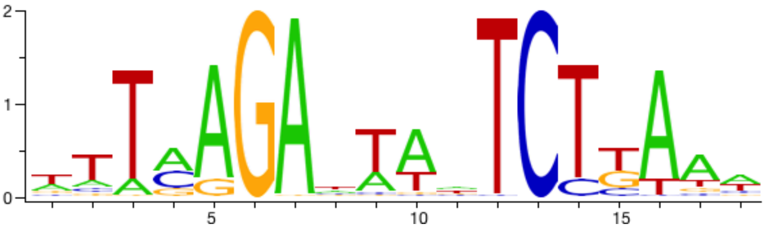

33. RhaS

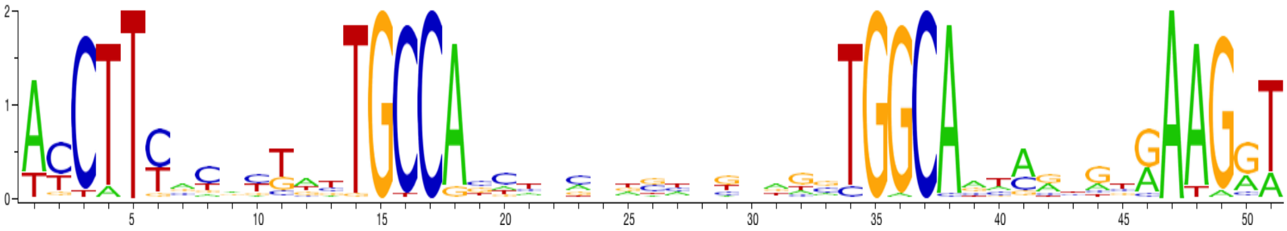

34. Rob

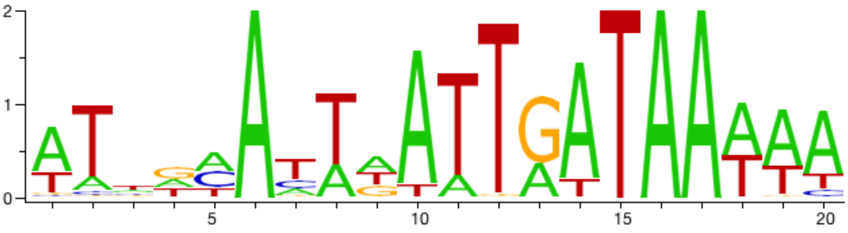

35. RpoE

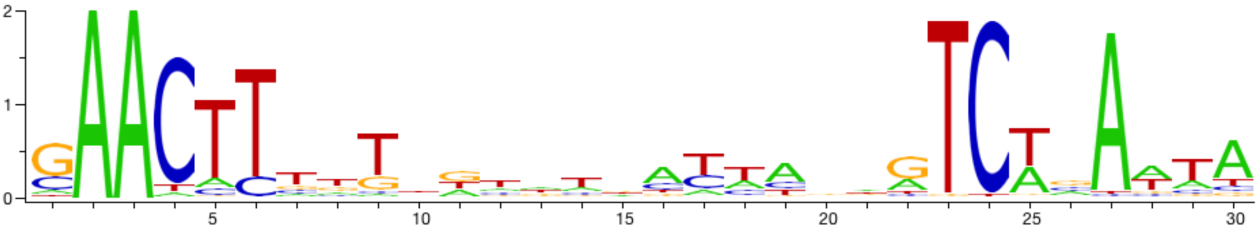

36. RpoH1

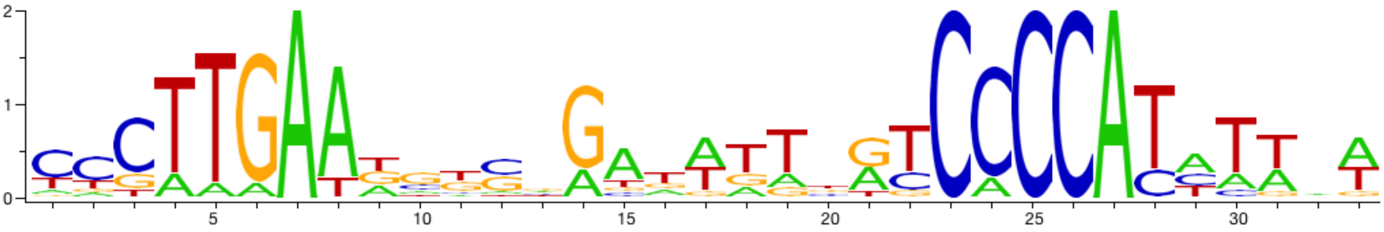

37. RpoH2

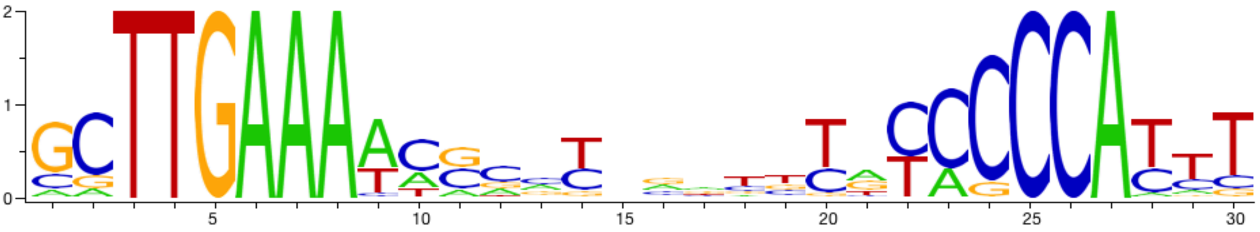

38. RpoN

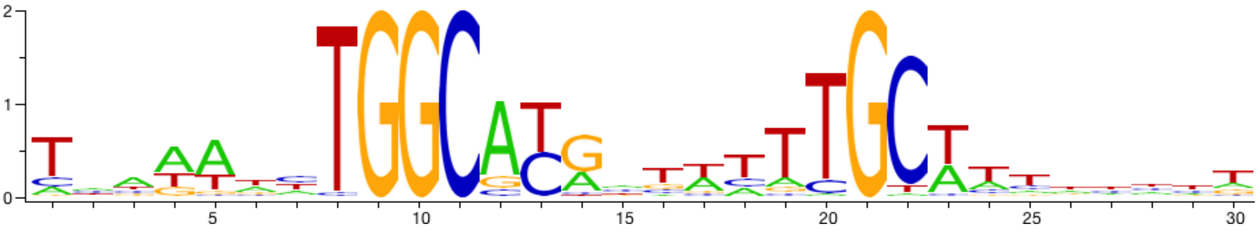

39. RtcR

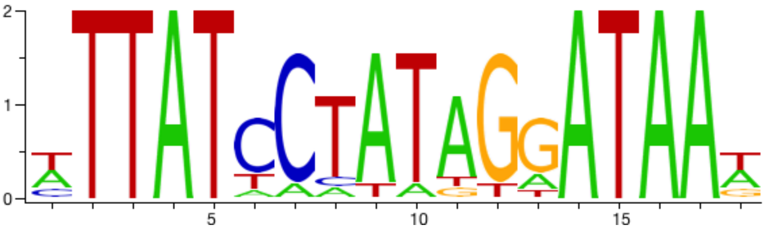

40. SlyA

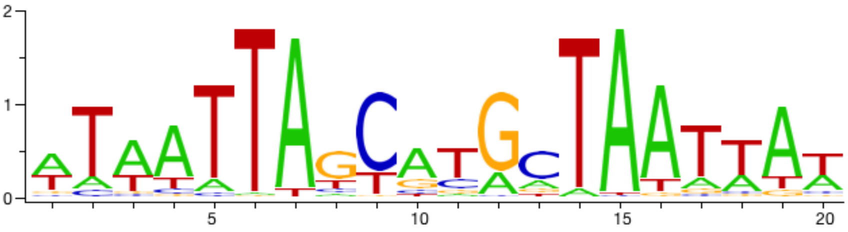

41. UxuR

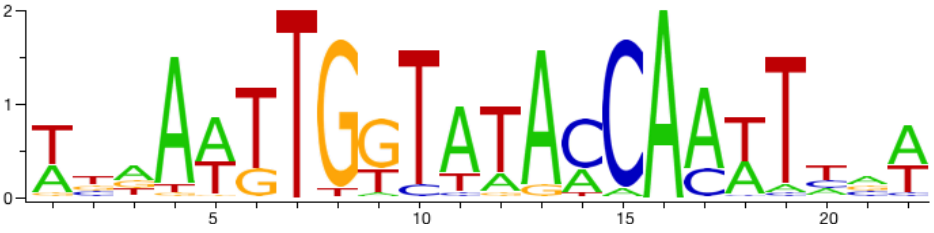

42. VasH

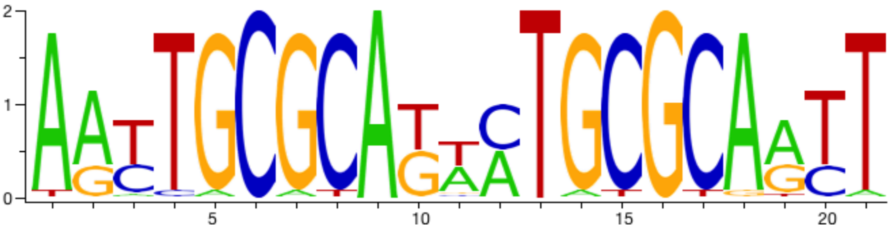

43. Zur

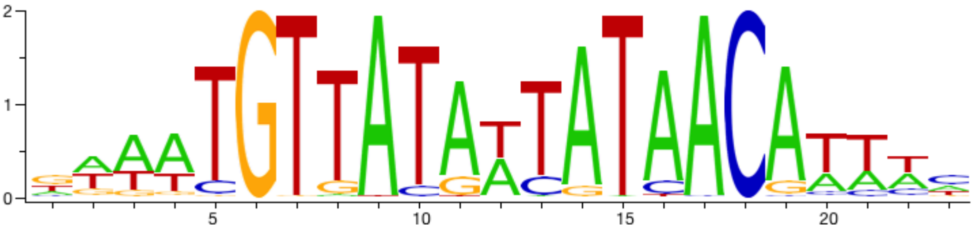

Supplement: Figure S2 [file peerj-04-2056-s006.pdf]
