# Supplementary material for: SigmoID: a user-friendly tool for improving bacterial genome annotation through analysis of transcription control signals
Source: PeerJ. 2016 May 24;4:e2056. doi: 10.7717/peerj.2056 (PMC4888284; doi:10.7717/peerj.2056)
Supplement: Supplementary file 7 [file peerj-04-2056-s002.odt]

| Regulatory protein | binding site length/ information content | number of binding sites used for profile construction | Source of the original binding site data |
| --- | --- | --- | --- |
| original | final |
| BirA | NA | 33/28.2 | 15 | RegPrecise |
| CscR | 20/28.6 | 6 | RegPrecise |
| FabR | 18/24.9 | 18/22.5 | 13 | RegPrecise |
| FliA | 27/23.5 | 31/20.7 | 72 | (Ide, Ikebe & Kutsukake, 1999) |
| FruR | 16/24.6 | 16 | RegPrecise |
| GlcC | 17/24.3 | 17/24 | 10 | RegPrecise |
| GntR | 20/24.4 | 20/24.1 | 32 | RegPrecise |
| HexR | NA | 21/31.5 | 35 | RegPrecise |
| HexR1 | 17/26.5 | 17/26.0 | 30 | RegPrecise |
| HrpL | NA | 29/20.7 | 51 | (Ferreira et al., 2006), AE016853.1* |
| HutC | 20/25.4 | 20/25.0 | 19 | RegPrecise |
| IscR | 25/34.7 | 15 | RegPrecise |
| LexA | 18/22.6 | 18/20.8 | 14 | RegPrecise |
| LexA2 | 16/31.0 | 16/28.6 | 5 | RegPrecise |
| LiuR | 18/26.6 | 18/26.2 | 32 | RegPrecise |
| LldR | 17/24.6 | 20 | RegPrecise |
| ModE | 24/30.3 | 24/27.4 | 9 | RegPrecise |
| NagQ | 18/29.3 | 18/29.2 | 4 | RegPrecise |
| NorR | 19/29.3 |  | 4 | RegPrecise |
| NrdR | 16/18.1 | 22/18.9 | 15 | RegPrecise |
| NrtR | 21/25.3 | 10 | RegPrecise |
| NtrC** | 17/20.5 | 17/18.9 | 18 | RegPrecise |
| PhhR | 20/21.3 | 20/23.0 | 32 | RegPrecise |
| PhnF | 12/24 | 3 | RegPrecise |
| PhnR | 12/19.9 | 16/23.6 | 7 | RegPrecise |
| PrpR | 12/21.1 | 12/20.3 | 16 | RegPrecise |
| PsrA | 20/23.1 | 20/22 | 69 | RegPrecise |
| PtxS | 20/30.3 | 16/28.3 | 10 | RegPrecise |
| PvdS | 30/25.9 | 29/26.8 | 12 | (Swingle et al., 2008) |
| RbsR | 14/27.3 | 15/27.1 | 5 | RegPrecise |
| RhpR | 16/18.4 | 18/19.1 | 14 | (Deng et al., 2010, 2014) |
| RpoH1 | 28/27.5 | 31/27.0 | 24 | (Nonaka et al., 2006) |
| RpoH2 | 29/24.7 | 32/30.4 | 11 | (Nonaka et al., 2006) |
| RpoN | 16/14.7 | 19/20.0 | 22 | (Barrios, Valderrama & Morett, 1999) |
| RutR** | 20/22.1 | 22/19.6 | 9 | RegPrecise |
| SahR | 20/38.6 | 20/36.9 | 16 | RegPrecise |
| VanR | 12/17.9 | 20/22.7 | 10 | RegPrecise |
| Zur** | 23/21.7 | 23/26.2 | 12 | RegPrecise |
