# Supplementary material for: SigmoID: a user-friendly tool for improving bacterial genome annotation through analysis of transcription control signals
Source: PeerJ. 2016 May 24;4:e2056. doi: 10.7717/peerj.2056 (PMC4888284; doi:10.7717/peerj.2056)
Supplement: Supplementary file 8 [file peerj-04-2056-s003.odt]

| TU No | Gene | Locus | Evidence | Final product annotation |
| --- | --- | --- | --- | --- |
| nhmmer score | Supported by RNA-Seq |
| 1 | sodA | GZ59_00980 | 12.7 | + | manganese superoxide dismutase |
| 2 | - | GZ59_04470 | 9.3 | + | hypothetical protein |
| 3 | osmY | GZ59_04860 | 10.5 | + | osmotically inducible protein Y |
| - | GZ59_04870 | putative membrane protein |
| - | GZ59_04880 | general stress response protein* |
| rihB | GZ59_04890 | TatD-family deoxyribonuclease |
| 4 | yjbJ* | GZ59_05480 | 13.6 | + | stress-induced protein* |
| 5 | gldA | GZ59_07810 | 10.4 | + | glycerol dehydrogenase |
| - | GZ59_07800 | putative membrane protein |
| - | GZ59_07790 | 4-oxalocrotonate tautomerase family enzyme |
| 6 | gltI | GZ59_13410 | 9.1 | + | glutamate/aspartate periplasmic binding protein |
| 7 | - | GZ59_15410 | 12.1 | + | sodium:dicarboxylate symporter |
| 8 | - | GZ59_15790 | 8.9 | + | hypothetical protein |
| prr* | GZ59_15800 | Gamma-aminobutyraldehyde dehydrogenase* |
| 9 | moeA | GZ59_17230 | 10.5 | - | molybdopterin biosynthesis protein |
| moeB | GZ59_17220 | molybdopterin biosynthesis protein |
| - | GZ59_17210 | hypothetical protein |
| 10 | ycdX* | GZ59_20370 | 13.1 | -** | alkaline phosphatase* |
| ycdY* | GZ59_20380 | chaperone protein YcdY* |
| 11 | - | GZ59_20890 | 14.9 | + | hypothetical protein |
| 12 | - | GZ59_20940 | 11.7 | + | hypothetical protein |
| 13 | - | GZ59_21230 | 12.7 | + | diguanylate cyclase/phosphodiesterase with PAS/PAC and GAF sensor |
| 14 | ychH* | GZ59_23780 | 13.6 | + | putative inner membrane stress-induced protein* |
| 15 | - | GZ59_24050 | 12.5 | + | putative metal-dependent peptidase |
| 16 | - | GZ59_27750 | 10.7 | + | hypothetical protein |
| 17 | ariR* | GZ59_28370 | 15.7 | + | Regulator of Acid Resistance* |
| 18 | elaB* | GZ59_30230 | 9.9 | + | ribosome-binding protein* |
| 19 | - | GZ59_31460 | 10.4 | + | PTS system, cellobiose-specific IIa component |
| 20 | yegS* | GZ59_32050 | 12.0 | + | Phosphatidylglycerol kinase, metal-dependent* |
| 21 | bsmB* | GZ59_39600 | 12.0 | -** | biofilm development protein* |
| 22 | ecnB | GZ59_39740 | 9.0 | + | ECN family pore-forming entericidin |
| - | GZ59_39730 | putative membrane protein |
| 23 | argD | GZ59_41210 | 14.7 | + | acetylornithine/succinyldiaminopimelate aminotransferase |
| 24 | occQ | GZ59_41370 | 10.1 | + | octopine transport system permease protein OccQ |
| occM | GZ59_41380 | octopine transport system permease protein OccM |
| occP | GZ59_41390 | octopine permease ATP-binding protein OccP |
