# Supplementary material for: SigmoID: a user-friendly tool for improving bacterial genome annotation through analysis of transcription control signals
Source: PeerJ. 2016 May 24;4:e2056. doi: 10.7717/peerj.2056 (PMC4888284; doi:10.7717/peerj.2056)
Supplement: Supplementary file 9 [file peerj-04-2056-s004.odt]

| locus_id | Regulator | Annotation produced by (or with the help of) |
| --- | --- | --- |
| Prokka | NCBI prokaryotic annotation pipeline | SigmoID |
| GZ59_30950 | SlyA | putative exported protein | sugar-binding protein | necrosis inducing protein |
| GZ59_11280 | SlyA | putative ABC transporter ATP binding protein | multidrug ABC transporter ATP binding protein | antimicrobial peptide efflux ABC transporter permease/ATPase |
| GZ59_42080 | SlyA, FNR | putative two-component system sensor kinase | sensor histidine kinase | Stationary phase sensor kinase regulating yjiY |
| GZ59_04720 | SlyA, ArcA | hypothetical protein | hypothetical protein | Type VI secretion system effector, Hcp1 family |
| GZ59_34530 | SlyA, Fur | hypothetical protein | hypothetical protein | type VI secretion protein TssK |
| GZ59_34520 | SlyA, Fur | putative membrane protein | chemotaxis protein MotB | type VI secretion protein TssL |
| GZ59_34460 | SlyA, Fur | hypothetical protein | membrane protein | type VI secretion-associated protein VasL |
| GZ59_35520 | RpoE | peptidase M23 | peptidase | Activator of AmiC, murein hydrolase activity |
| GZ59_07400 | RpoE | hypothetical protein | hypothetical protein | Transcriptional regulator, AbiEi antitoxin, Type IV TA system |
| GZ59_07410 | RpoE | hypothetical protein | hypothetical protein | Nucleotidyl transferase AbiEii toxin, Type IV TA system |
| GZ59_24260 | KdgR | putative exported protein | porin | KdgM-like oligalacturonate-specific porin |
| GZ59_04140 | KdgR | hypothetical protein | hypothetical protein | toxin-antitoxin biofilm protein TabA |
| GZ59_01110 expR | KdgR, Fur | transcriptional regulator, LuxR family | LuxR family transcriptional regulator | transcriptional activator protein ExpR |
| GZ59_26860 | RpoN | hypothetical protein | urea carboxylase | urea carboxylase-associated protein 1 |
| GZ59_26870 | RpoN | hypothetical protein | urea carboxylase | urea carboxylase-associated protein 2 |
| GZ59_26880 | RpoN | ABC transporter related | lauroyl acyltransferase | urea carboxylase-associated ABC transporter, ATPase component |
| GZ59_26890 | RpoN | ABC transporter, permease protein | lipid kinase | urea carboxylase-associated ABC transporter, permease component |
| GZ59_26900 | RpoN | putative ABC transporter, periplasmic protein | lipid kinase | urea carboxylase-associated ABC transporter, periplasmic substrate-binding component |
| GZ59_43470 | RpoN, VasH | hypothetical protein | hypothetical protein | type VI secretion system effector, Hcp1 family |
| GZ59_24880 | HrpL | pectate lyase | pectate lyase | HrpW harpin |
| GZ59_21690 | Fur | hypothetical protein | hypothetical protein | putative manganese efflux pump MntP |
| GZ59_33530 | Fur | ABC transporter ATP biding protein | sugar ABC transporter | Ferric iron (Fe3+) ABC transporter, ATP-binding protein |
| GZ59_33540 | Fur | ABC transporter, permease protein | ABC transporter permease | Ferric iron (Fe3+) ABC transporter, permease protein |
| GZ59_21950 | CRP, ArcA | DNA polymerase III, theta subunit | DNA polymerase III subunit theta | Uncharacterised protein |
| GZ59_03880 | CpxR (KdgR, CRP) | substrate-binding periplasmic protein | C4-dicarboxylate ABC transporter | TRAP transporter periplasmic substrate binding subunit DctP |
| GZ59_03870 | CpxR (KdgR, CRP) | putative membrane protein | C4-dicarboxylate ABC transporter | TRAP transporter permease subunit DctQ |
| GZ59_03860 | CpxR (KdgR, CRP) | putative DedA-family membrane protein | membrane protein | TRAP transporter permease subunit DctM |
| GZ59_28390 yccA | CpxR | putative membrane protein | membrane protein | Modulator of FtsH protease, inner membrane protein |
